# Design framework and optimization of portable biomedical waste decomposition systems using ANN and MOPSO

**DOI:** 10.1038/s41598-025-33723-y

**Published:** 2025-12-25

**Authors:** Naresh N. Bhaiswar, Sushant S. Satputaley, Sandeep M. Kadam, P. Dinesha, Sooraj Mohan

**Affiliations:** 1 Post Graduate Teaching Departments (PGTD) of Electronics & Computer Science, Rashtrasant Tukadoji Maharaj Nagpur University, Nagpur, 440033 Maharashtra India; 2 Department of Mechanical Engineering, Priyadarshini College of Engineering, Nagpur, 440019 Maharashtra India; 3 Department of Mechanical Engineering, St. Vincent Pallotti College of Engineering & Technology, Nagpur, Maharashtra India; 4 Thermax Limited, Pune, Maharashtra India; 5 Department of Mechanical and Industrial Engineering, Manipal Institute of Technology, Manipal Academy of Higher Education, Manipal, 576104 India

**Keywords:** BMW, Energy optimization, Machine learning, Sustainable incineration, Climate action, Energy science and technology, Engineering, Environmental sciences, Materials science, Mathematics and computing

## Abstract

Biomedical waste (BMW) incineration requires accurate prediction of energy demand and efficiency due to its heterogeneous composition. In this study, material and energy balance calculations were combined with design of experiments (DOE), analysis of variance (ANOVA), artificial neural network (ANN) modeling, and multi-objective particle swarm optimization (MOPSO). The novelty of the work presents the integration of metaheuristic optimization (MOPSO) into the biomedical waste incineration process. Results showed cellulose content as the most significant determinant of auxiliary energy requirement, with higher cellulose reducing LPG demand, while tissue and moisture exerted secondary but measurable effects. Efficiency ranged between 95.5 and 95.6%, with efficiency decreasing at higher moisture levels. The ANN model achieved near-perfect prediction accuracy (R² > 0.9999), enabling robust surrogate-based optimization. MOPSO analysis identified Pareto-optimal operating conditions where auxiliary energy demand reduced from 99.7 MJ/h to 97.2 MJ/h while efficiency improved from 95.52% to 95.60%. Under optimal waste composition identified by the ANN-MOPSO hybrid, auxiliary LPG consumption reduced from 33.8 to 27.4 kg/h, indicating strong potential for energy savings within the studied domain.

## Introduction

Safe management of biomedical waste (BMW) is increasingly recognised as a global challenge, directly linked to the United Nations Sustainable Development Goals (SDGs)^1^, particularly SDG 3 (Good Health and Well-being), SDG 6 (Clean Water and Sanitation), SDG 11 (Sustainable Cities and Communities), and SDG 12 (Responsible Consumption and Production). Effective BMW treatment is critical to protecting human health, reducing infection risk, and ensuring environmental sustainability. The rapid expansion of healthcare facilities, rural healthcare outreach, population growth, and pandemic responses have significantly increased BMW generation worldwide^2^.

Biomedical or clinical waste includes used needles and syringes, contaminated dressings, pharmaceuticals, body fluids, and laboratory disposables. Unmanaged BMW poses a severe threat to public health and the environment. Healthcare staff, waste handlers, and the community are vulnerable to infection from pathogens, while unregulated landfilling and open burning release toxic compounds, contaminate soil and water, and contribute to the spread of antibiotic-resistant bacteria^3^. The risks are even greater in low-resource and rural areas, where the absence of waste treatment infrastructure prevents safe management^4^.

Centralised BMW treatment facilities are often scarce or unavailable in rural and remote regions, disaster zones, and temporary healthcare facilities such as mobile hospitals and field camps^5^. Conventional methods such as large-scale incinerators and autoclaves require stable infrastructure, electricity supply, and skilled personnel, which are typically lacking in these contexts^6^. As a result, there is a growing demand for decentralised, effective, and safe BMW treatment solutions that are adaptable to resource-limited conditions. Portable decomposition systems can play a key role in this regard^7^. Compact, mobile, and easy-to-use, they enable on-site management of hazardous BMW, thereby minimising infection and contamination risks and eliminating unsafe and costly long-distance waste transportation. In temporary healthcare settings deployed during pandemics, natural disasters, or conflicts, such portable systems ensure real-time, hygienic, and regulatory-compliant waste disposal. This enhances public health safety, protects frontline workers, and reduces untreated waste release into the environment. Furthermore, such systems contribute to achieving universal health coverage, disaster preparedness, and sustainable healthcare practices in line with global sustainability goals^8^.

Despite advances in healthcare infrastructure, BMW management systems in decentralised or low-resource contexts face several limitations. Most existing systems are designed for centralised, large-scale operations that depend on extensive logistics, trained staff, and a reliable energy supply^9^. Conventional treatment systems involve high capital and operating costs, including expenditure on equipment, skilled labour, fuel, and emissions control systems, which smaller healthcare units cannot afford^10^. Inefficient incineration can emit dioxins, furans, and heavy metals, while untreated waste leachates contaminate soil and groundwater. Transporting BMW to distant treatment facilities poses biohazard risks, increases carbon footprint, and delays safe disposal^11^. Poor waste segregation and the absence of real-time monitoring often lead to inefficiencies and violations of environmental standards. Stationary systems are unsuitable for emergency settings, disaster zones, or mobile medical camps where rapid on-site treatment is essential^12^.

To overcome these restrictions, innovative, portable, and flexible BMW treatment systems are needed, with minimal infrastructure requirements and reduced environmental impacts. A scientifically designed portable decomposition unit based on thermodynamic and material flow principles can provide a sustainable solution for these health and environmental concerns.

The main BMW decomposition methods include incineration, autoclaving, and pyrolysis^13^, each with specific strengths and weaknesses. High-temperature incineration reduces waste volume and destroys pathogens, but emits toxic pollutants without proper emission controls^14^. Autoclaving, which uses high-pressure saturated steam, effectively sterilises sharps and microbiological waste but is unsuitable for pathological or pharmaceutical waste and does not reduce waste volume. Pyrolysis, the thermal decomposition of waste in the absence of oxygen, can generate gas, oil, and char as by-products. It is more energy-efficient and environmentally benign than incineration, but its complexity and high cost hinder adoption in low-resource areas^15^. While these methods are effective in controlled environments, they are often unsafe, impractical, or economically unfeasible in rural and decentralised healthcare settings, underscoring the need for flexible alternatives.

Design and optimisation of BMW management systems require the application of heat and material balance principles. Material balance allows engineers to track all system inputs, outputs, and accumulations, ensuring proper throughput and reducing hazardous by-products^16^. Inputs typically include waste, combustion air, and auxiliary chemicals such as water or catalysts, while outputs consist of flue gases, solid residues, and effluents^17^. Heat balance accounts for the energy required to initiate and sustain thermal decomposition reactions, including combustion heat, conductive and radiative losses, and the energy needed to raise waste to operational temperatures (commonly > 800 °C for incineration or 500 °C for pyrolysis). Such balances optimise fuel usage, improve insulation, and guide reactor sizing, emission controls, and energy recovery. When combined with computational simulations, they enable accurate prediction of thermal behaviour and system performance before physical prototyping, thereby reducing costs and improving design dependability.

Portable BMW management devices offer practical advantages for decentralised healthcare delivery. They allow real-time waste neutralisation at rural clinics, vaccination drives, or mobile hospitals, where centralised facilities are inaccessible. Units with capacities ranging from 1 to 5 kg/h to 10 to 20 kg/h can be rapidly deployed and scaled as needed^18^. Modern systems also incorporate emission control mechanisms and energy recovery features, enhancing environmental sustainability. However, several challenges remain. Portable units are often unsuitable for high-volume waste loads; regular maintenance and calibration are required, and dependence on electricity, diesel, or gas may restrict their use in remote or disaster-stricken locations. Furthermore, most existing systems lack flexibility, scalability, or adaptation to varying BMW compositions, and very few studies address comprehensive life cycle impacts such as greenhouse gas emissions or dioxin release.

Recent advancements in computational intelligence have enabled the application of machine learning methods in process modelling and optimization across thermal and environmental systems^19–22^. These data-driven techniques can efficiently capture nonlinear relationships between multiple input variables and process responses, which are often difficult to represent through conventional analytical models. Among various learning approaches, artificial neural networks (ANNs) have shown particular promise in modelling complex combustion and incineration phenomena due to their high adaptability and predictive capability^23–25^. ANN has been successfully implemented in bio-waste adsorbents^26^, water desalination^27^, enzyme extraction from mushroom wastes^28^, energy storage systems^29^, etc. When coupled with optimization algorithms, such models can serve as powerful surrogate frameworks for identifying energy-efficient operating conditions.

Recent studies have demonstrated the growing application of hybrid data-driven frameworks for process optimization in energy and emission systems. An ANN-based optimization study was employed for low-carbon hydrogen production via sorption-enhanced steam methane reforming, achieving a 15% cost reduction compared to conventional SMR^30^. Similar Pearson correlation-ANN approaches have been applied to ammonia removal^31^ and shipboard CO_2_ capture, significantly improving computational efficiency and energy use^32^. While these works highlight the promise of machine learning-assisted optimization, their focus remains on gaseous process systems rather than heterogeneous solid-waste incineration, which is addressed in the present study. Integrating such machine learning tools ensures a more robust, systematic understanding of biomedical waste treatment processes, thereby improving design and operational performance. The integration of ANN with Particle Swarm Optimization (PSO) is primarily used in waste and energy systems for enhanced prediction, modeling, and optimization of complex, non-linear processes. This hybrid approach leverages the ANN’s ability to model complex relationships and the PSO’s efficiency in finding global optimum solutions to improve performance and decision-making in various applications^33^.

From the literature, it is clear that portable BMW treatment technologies hold strong potential, but critical gaps remain. Current systems lack integration of thermodynamic principles into design, show limited energy efficiency and emission control in off-grid settings, and often fail to consider real-world variations in BMW composition. Additionally, research seldom combines experimental, simulation-based, and optimisation approaches to enhance system performance.

The present study aims to address these gaps by developing a theoretical design framework for a portable BMW decomposition system based on heat and material balance principle**s**. The specific objectives are:

- to characterise BMW composition and variability in decentralised healthcare settings,
- to estimate thermal energy requirements for efficient decomposition,
- to model material flows and predict output by-products, and.
- to develop machine learning models and optimise the best waste conditions for minimising energy requirements.

The novelty of the work emphasises the integration of metaheuristic optimization techniques like particle swarms to the biomedical waste incineration process. By integrating these aspects, the study contributes to the advancement of sustainable, decentralised BMW management technologies that align with public health needs and global sustainability targets.

## Methods

Quantitative assessment of the incinerator was carried out using a comprehensive heat and material balance framework. This technique estimates incinerator input and output conditions, determines combustion air and auxiliary fuel needs for medical waste, and assesses existing incinerator restrictions for known waste. The methodological approach followed in this study integrates experimental data collection, thermodynamic analysis, and computational modeling to evaluate and optimize the BMW incineration process. The workflow consists of:

(i) characterization of representative BMW composition from healthcare facilities;
(ii) development of a comprehensive material and energy balance to estimate combustion requirements and system efficiency;
(iii) structured variation of waste composition through a design of experiments (DOE);
(iv) statistical analysis of the results using ANOVA to identify key influencing factors;
(v) development of an artificial neural network (ANN) model to predict energy demand and efficiency; and.
(vi) multi-objective optimization using particle swarm optimization (MOPSO) to determine operating conditions that minimize energy use while maximizing efficiency.

This stepwise framework provides a systematic linkage between experimental data, modeling, and optimization to ensure a reproducible and comprehensive analysis. While several modeling and optimization approaches have been applied to incineration and thermal treatment systems, most rely on simplified empirical correlations or single-objective formulations that cannot fully capture nonlinear interactions among waste constituents and operating variables. Conventional models also struggle to simultaneously predict performance and guide optimization. The integrated ANN-MOPSO framework adopted in this study addresses these gaps by combining data-driven learning with multi-objective optimization, enabling more robust prediction and efficient decision-making within the defined design space.

### BMW characterization

Characterization of biomedical waste has been extensively studied in the literature. Fuel consumption and cycle time are measured during the incineration. These data serve as a baseline for comparing liquefied petroleum gas (LPG) usage in the incineration of larger waste quantities. Fundamental parameters such as energy balance calculations, auxiliary fuel consumption, LPG utilization, cycle time, ash content, and partially burned residues will be compared with future observations. Such comparisons are essential to evaluate overall system efficiency. For the present study, the PPE-clad research team visited the Common Biomedical Waste Treatment and Disposal Facility (CBWTF) in different wards at Government Medical College, Nagpur, India, where the bags were unwrapped and the biomedical waste components were segregated for 10 kg of waste. The bags, which ranged in weight from 4 kg to 10 kg, were gathered from various wards. One or two bags were collected for segregation, and their combined weight was 10 kg. The approximate compositional ranges, along with the characteristics of the different elements, are presented in Table 1. The range of the elemental composition is based on the segregated elements from the unwrapped bags, and the corresponding elemental characteristics are taken from^34^.

**Table 1:** Elemental composition of BMW.

Elements	% composition in BMW	Chemical formula	Molecular Weight (g/mol)	Gross Heating Value (kJ/kg)
Cellulose, swabs	10–31	C_6_H_10_O_5_	162.1	18,568
Plastics-Poly Ethylene	2–5	(C_2_H_4_)_x_	28.1	46,340
PVC	2–4	(C_2_H_3_Cl)_x_	62.5	22,630
Tissue	20–40	C_5_H_10_O_3_	118.1	20,471
Moisture	20–40	H_2_O	18	-
Ash	8–10	-	-	-

### Estimation of energy requirement

A comprehensive material and energy balance framework was developed to evaluate the combustion of BMW. The process begins with the characterization of feedstock, where the major combustible constituents are identified. Based on these compositions, stoichiometric combustion reactions are formulated for each component to determine the theoretical oxygen requirement as shown in reactions R1 through R4.

Tissue - C_5_H_10_O_3_ + 6O_2_ = 5CO_2_ + 5H_2_O (R1).

Polyethylene- (C_2_H_4_)_x_ + 3O_2_ = 2CO_2_ + 2H_2_O (R2).

PVC − 2(C_2_H3Cl)_x_ +5O_2_ = 4CO_2_ + 2H_2_O + 2HCl (R3).

Cellulose - C_6_H_10_O_5_ + 6O_2_ = 6CO_2_ + 5H_2_O (R4).

For a generic component, the combustion reaction can be expressed as shown in equation (R5).

C_x_H_y_O_z_+ aO_2_→ bCO_2_+ cH_2_ O + dHCl (R5)

The total stoichiometric oxygen requirement is obtained by summing the contributions from all components as shown in Eq. (1).

1 $$\:{O}_{2\_stoich}={\sum\:}_{i}\left(moles\:of\:{O}_{2}\:per\:mole\:of\:component\right)\times\:\left(mass\:flow\:of\:component\right)$$

The theoretical air required for combustion is calculated from the stoichiometric oxygen, taking into account the oxygen fraction in air, $\:{X}_{{O}_{2}}$​​, using Eq. (2).

2 $$\:{A}_{th}=\frac{{O}_{2\_stoich}}{{X}_{{O}_{2}}}$$

A 50% excess air is assumed for complete combustion, and the total air requirement is calculated.

Mass balance is then performed by accounting for all input streams, including waste, inherent moisture, and supplied air, yielding the total mass input. The corresponding outputs consist of flue gases, moisture, acid gases such as HCl from chlorinated compounds, and residual ash. The energy balance is constructed to quantify heat generation and distribution within the system. The energy input from the waste is determined using its calorific value as shown in Eq. (3).

3 $$\:{Q}_{w}={m}_{w}{CV}_{w}$$

The energy output is distributed among several pathways such as Heat absorbed by flue gases, Moisture present in the waste and air absorbs both sensible and latent heat, Heat absorbed by ash, and the radiative loss that is taken as a fraction (5%) of the total energy.

If the cumulative energy output exceeds the energy provided by the waste, the deficit ($\:{Q}_{deficit}$) is compensated using an auxiliary fuel. The fuel considered in the study is liquefied petroleum gas (LPG) with a GCV of 46,100 kJ/kg. The required mass of fuel is calculated using Eq. (4).

4 $$\:{m}_{fuel}=\frac{{Q}_{deficit}}{{CV}_{fuel}}$$

The overall thermal efficiency of the system is determined as the ratio of energy output ($\:{Q}_{o}$) (considering all the heat transfers to moisture, ash, flue gas, etc.) to the total energy input (including contributions from both the waste ($\:{Q}_{w}$) and auxiliary fuel $\:{Q}_{fuel}$) as shown in Eq. (5).

5 $$\:\eta\:=\frac{{Q}_{o}}{{Q}_{w}+{Q}_{fuel}}$$

### Design of experiments

The thermal behavior of BMW during incineration was strongly dependent on its heterogeneous composition. To systematically investigate the influence of different waste constituents on combustion performance, a design of experiments (DOE) framework was employed. The DOE enabled structured variation of input factors within realistic ranges while minimizing the number of experimental runs required for statistical evaluation.

Six independent factors were selected based on their prevalence in BMW streams and their potential impact on thermal characteristics: cellulose, plastics (polyethylene), PVC, tissue, moisture, and ash. Each factor was expressed as a percentage of the total feed mass. The ranges of variation for each constituent were derived from compositional analyses of representative hospital waste samples and literature-reported bounds.

The experimental matrix was constructed using a factorial-type design with constrained mixtures, ensuring that the sum of component fractions equaled 100%. Levels of cellulose, plastics, PVC, and tissue were systematically varied across low, medium, and high proportions, while moisture and ash contents were adjusted to reflect operational variability in feed preparation and handling. A total of 48 unique runs were generated, covering a wide spectrum of realistic BMW compositions. The DOE matrix is summarized in Table 2.

**Table 2:** Factorial design of experiments followed in this work.

Cellulose	Plastics	PVC	Tissue	Moisture	Ash
10	2	2	36	40	10
10	2	2	38	40	8
10	2	2	40	36	10
10	2	2	40	38	8
10	2	4	34	40	10
10	2	4	36	40	8
10	2	4	40	34	10
10	2	4	40	36	8
10	5	2	33	40	10
10	5	2	35	40	8
10	5	2	40	33	10
10	5	2	40	35	8
10	5	4	31	40	10
10	5	4	33	40	8
10	5	4	40	31	10
10	5	4	40	33	8
21	5	4	20	40	10
21	5	4	40	20	10
23	5	2	20	40	10
23	5	2	40	20	10
23	5	4	20	40	8
23	5	4	40	20	8
24	2	4	20	40	10
24	2	4	40	20	10
25	5	2	20	40	8
25	5	2	40	20	8
26	2	2	20	40	10
26	2	2	40	20	10
26	2	4	20	40	8
26	2	4	40	20	8
28	2	2	20	40	8
28	2	2	40	20	8
31	2	2	20	35	10
31	2	2	20	37	8
31	2	2	35	20	10
31	2	2	37	20	8
31	2	4	20	33	10
31	2	4	20	35	8
31	2	4	33	20	10
31	2	4	35	20	8
31	5	2	20	32	10
31	5	2	20	34	8
31	5	2	32	20	10
31	5	2	34	20	8
31	5	4	20	30	10
31	5	4	20	32	8
31	5	4	30	20	10
31	5	4	32	20	8

Each run in the DOE provided a defined waste composition, which was then used as the input for incineration performance evaluation. Response variables included auxiliary energy requirement, auxiliary LPG consumption, and overall thermal efficiency of the incinerator. The DOE allowed for subsequent application of analysis of variance (ANOVA) to quantify the relative contribution of each factor and to identify statistically significant drivers of energy demand and efficiency.

### Analysis of variance (ANOVA)

To evaluate the influence of BMW composition on combustion performance, ANOVA was employed. The objective was to quantify the extent to which individual waste components contributed to variations in (i) auxiliary energy requirement, (ii) auxiliary LPG consumption, and (iii) overall system efficiency.

The independent factors considered were six major compositional constituents of BMW: cellulose swabs, plastics (polyethylene), PVC, tissue, moisture, and ash. Each factor was defined in terms of its percentage contribution to the total waste feed, bounded by experimentally observed ranges. The response variables were calculated for auxiliary energy requirement (kJ/h), LPG consumption rate (kg/h), and thermal efficiency. ANOVA was selected because it allows partitioning of the total variance in each response into contributions attributable to each factor (between-group variation) and the experimental error (within-group variation). For each output, one-way ANOVA was performed separately for all six factors using the ordinary least squares (OLS) approach in the *statsmodels* package (Python 3.12). Each waste component was treated as a categorical predictor, and F-tests were used to determine statistical significance at a 95% confidence level.

It should be noted that the six compositional variables (cellulose, plastics, PVC, tissue, moisture, and ash) are expressed as percentages that sum to 100%. As a result, they are not statistically independent and exhibit inherent multicollinearity. In this study, ANOVA is therefore used in an exploratory manner to screen for dominant trends rather than to provide strictly independent effect estimates. Interaction effects between components are not explicitly modeled and cannot be fully separated from the multicollinearity induced by the mixture constraint.

### Artificial neural network modeling

**Fig. 1:**
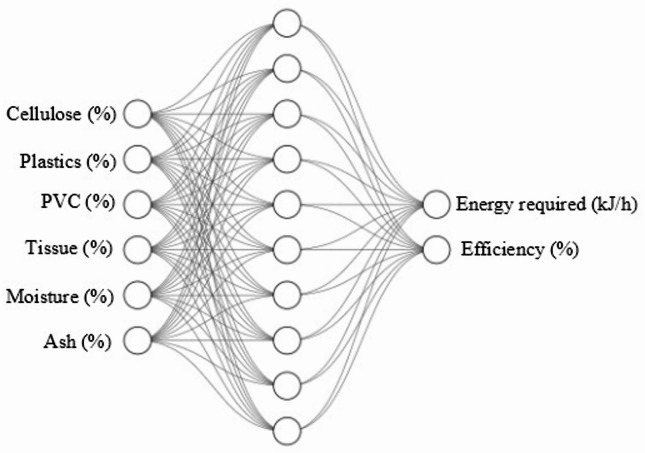
Neural network architecture followed in this work.

In the present study, the prediction of energy requirement and operational efficiency of the medical waste incineration system was carried out using an artificial neural network (ANN) coupled with response surface contour analysis. The ANN modelling was adopted as it captures nonlinear correlations effectively without requiring an explicit mathematical formulation of the incineration process. The ANN model architecture and hyperparameters were optimized through iterative testing to ensure accurate prediction and stable convergence. The final network comprised a single hidden layer with ten neurons, which provided the best balance between accuracy and computational simplicity. 10 neurons were selected after evaluating architectures ranging from 5 to 20 neurons. Beyond 10 neurons, the improvement in R2 was negligible, while model complexity and training time increased. The tangent-sigmoid activation function was used in the hidden layer and a linear function in the output layer. The model was trained using the Levenberg-Marquardt algorithm, which adapts the learning rate internally for rapid convergence. Data were divided into training (70%), validation (15%), and testing (15%) subsets. Training was automatically terminated by early stopping when validation error failed to decrease for six consecutive iterations. Mean squared error was used as the performance metric. This configuration yielded consistent results with high predictive accuracy and no signs of overfitting. The network architecture consisted of an input layer with 6 process parameters (cellulose, plastic, PVC, tissue, moisture, and ash), a single hidden layer with 10 neurons, and an output layer representing 2 responses, energy requirement (kJ/h) and efficiency (%). The network was retrained until the coefficient of determination for training, validation, and testing was more than 0.99. The neural network architecture used in the study is represented in Fig. 1.

Following successful ANN modeling, the trained weights and biases were extracted and implemented in Python to generate response surface contour plots. This approach enabled visualization of the combined effect of the most dominant factors identified through ANOVA, namely cellulose, tissue, and moisture contents, on the energy requirement and efficiency of the system. Two-dimensional contour maps were constructed, where moisture-cellulose interaction was studied with respect to energy demand and efficiency. These contour surfaces provide a clear graphical representation of process behavior, aiding in the identification of optimum operating conditions for the incineration process. All ANN predictions and contour surfaces were generated within the compositional bounds defined by the design of experiments, ensuring that the model operated strictly within its trained and validated domain without extrapolation beyond experimental limits.

### Multi-objective optimization using swarm intelligence

In this study, a multi-objective particle swarm optimization (MOPSO) algorithm was implemented in MATLAB 2024b to optimize the operating conditions of the medical waste incineration system using the ANN model as the surrogate evaluator. Particle swarms are the most versatile, simple, and fast algorithm for multi-objective problems, and hence MOPSO was adopted due to its population-based, stochastic nature that enables exploration of a wide solution space and simultaneous handling of multiple objectives. PSO has been used to optimize operating conditions in various thermal systems like IC engines^35^, solar systems^36^, biofuels^37^, nanofluids^38^, etc. The optimization aimed to simultaneously minimize the auxiliary energy requirement and maximize the efficiency of the system by varying six input parameters, namely cellulose, plastics, PVC, tissue, moisture, and ash. During optimization, the sum of all compositional fractions (cellulose, plastics, PVC, tissue, moisture, and ash) was constrained to 100%. At each iteration, particle positions were normalized to ensure strict adherence to this mixture limit, preventing infeasible solutions.

The optimization framework employed a swarm population size of 200 particles, with each particle representing a potential solution vector in the six-dimensional decision space. The literature suggests using a swarm size of 25 times the number of process parameters to avoid false minima^39^. Hence, the swarm size has been kept at 200. A repository size of 200 was maintained to store non-dominated solutions, ensuring diversity in the Pareto front. As the initial set of trials showed that the convergence of the solutions happened at 20 to 25 generations, the maximum number of generations has been kept at 30, during which particles updated their positions and velocities according to both individual and social learning components. The inertia weight was fixed at 0.5, while the cognitive and social confidence factors were both set to 2, providing a balanced trade-off between exploration of the solution space and exploitation of promising regions.

To enhance the diversity of solutions and prevent premature convergence, the external repository was divided into 20 hypercubes per dimension, guiding the leader selection process. The maximum particle velocity was restricted to 40% of the decision variable range, thereby avoiding excessive oscillations in particle movement. A uniform mutation probability of 0.5 was incorporated to further improve the global search ability and maintain diversity in the population.

It is important to note that the entire analysis in this study is based on theoretical material and energy balance simulations. These calculations enable a structured understanding of combustion behaviour and optimization feasibility, but do not replicate real combustion dynamics, emissions, or operational variability.

## Results and discussion

Design of Experiments was implemented in this work to determine the energy and efficiency of the BMW incinerator. The outputs of energy required (kJ/h), fuel required (kg/h), and the machine efficiency were calculated as per the DOE table. The obtained results were further analysed for ANOVA to identify the most significant parameter affecting the output. Furthermore, the ANN model was fit to the dataset, which was further optimized using swarm intelligence to ascertain the optimal conditions for minimum energy requirement and maximum efficiency.

### ANOVA

The combustion characteristics of BMW were statistically analyzed using ANOVA to determine the influence of individual waste components of cellulose, plastics (polyethylene), PVC, tissue, moisture, and ash on auxiliary energy demand, auxiliary LPG consumption, and overall efficiency.

#### Auxiliary energy requirement

ANOVA revealed that cellulose and tissue fractions were the most significant contributors to variations in auxiliary energy demand (*p* < 0.001), with F-values of 33.79 and 6.81, respectively. Higher cellulose content reduced the auxiliary energy requirement, consistent with its relatively high volatile matter and ease of combustion, which enhances the self-sustaining capacity of the waste matrix. Conversely, tissue, despite also being cellulosic, displayed heterogeneity in its moisture retention and density, leading to statistically significant but less pronounced effects compared to pure cellulose swabs. Plastics (polyethylene), PVC, moisture, and ash fractions were not statistically significant (*p* > 0.05), indicating that within the tested ranges, their variation did not strongly alter the external energy needed to sustain combustion. This outcome suggests that the base calorific contribution of plastics was sufficient to offset their variation, while PVC and ash acted more as inert diluents rather than active determinants of fuel support.

#### Auxiliary LPG requirement

Patterns similar to energy demand were observed for auxiliary LPG consumption. Cellulose content again emerged as the dominant factor (F = 33.79, *p* < 0.001), followed by tissue fraction (F = 6.81, *p* < 0.001). A higher cellulose fraction reduced LPG requirement, consistent with its contribution to combustion stability and thermal output. The tissue fraction, however, increased fuel consumption at higher levels, reflecting the latent heat burden associated with its higher bound water content. Other components like polyethylene, PVC, moisture, and ash did not exert a significant influence. The non-significance of moisture content (*p* = 0.13) in this case suggests that although high moisture fraction increases latent heat loss, its effect was masked by the dominating role of cellulose and tissue in governing the energy balance. These results indicate that optimizing the proportion of combustible fibrous fractions can directly reduce reliance on external LPG during BMW incineration.

#### Efficiency

For efficiency, both cellulose (F = 12.72, *p* < 0.001) and moisture (F = 21.59, *p* < 0.001) were identified as statistically significant factors. Higher cellulose fractions were associated with improved efficiency, attributable to enhanced flame stability and uniform burning. In contrast, higher moisture content significantly reduced efficiency, as energy was diverted towards evaporation rather than oxidation of organics, leading to incomplete combustion and lower thermal efficiency. Tissue fraction showed marginal significance (*p* = 0.06), suggesting that its variable composition can influence efficiency under certain operating conditions. Plastics, PVC, and ash did not significantly affect efficiency (*p* > 0.2), implying that while plastics provide calorific input, their relatively narrow concentration range in BMW did not alter overall efficiency. Table 3 shows the ANOVA table considering all three output variables.

**Table 3:** ANOVA analysis conducted for energy requirement, LPG fuel requirement, and machine efficiency.

Factor	Auxiliary Energy		LPG required		Efficiency	
	F	*p*-value	F	*p*-value	F	*p*-value
Cellulose	33.79121	**7.36E-15**	33.79121	**7.36E-15**	12.72264	**1.85E-08**
Plastics_PE	1.086973	0.302591	1.086973	0.302591	1.189824	0.281046
PVC	1.546827	0.219906	1.546827	0.219906	0.995351	0.323657
Tissue	6.81402	**6.47E-06**	6.81402	**6.47E-06**	2.016533	0.059679
Moisture	1.647462	0.131382	1.647462	0.131382	21.58876	**1.6E-12**
Ash	0.607113	0.439867	0.607113	0.439867	5.05E-06	0.998216

Collectively, these findings highlight cellulose as the primary determinant of both auxiliary fuel requirement and efficiency in BMW combustion. Tissue fractions, although cellulosic, impose a higher energy penalty due to their variable moisture-binding nature. Moisture content, while not critical in determining auxiliary LPG consumption, strongly influences efficiency, underlining the need for pre-drying or thermal conditioning of BMW prior to incineration. The limited influence of plastics and PVC reflects their calorific stability at the tested levels, while the ash fraction behaved inertly, diluting the effective combustible fraction without statistically significant effects.

From a practical standpoint, enhancing the cellulose-to-moisture ratio in the waste stream reduces auxiliary LPG use and improves efficiency. This aligns with sustainability goals by lowering fossil fuel dependency in BMW incineration. Optimized segregation strategies (e.g., reducing high-moisture tissue fractions before incineration) can thus serve as a direct intervention for energy-efficient and environmentally responsible waste management. The performance findings (auxiliary energy requirement, fuel use, and efficiency) must be interpreted within the context of a simulation-based evaluation. These results provide theoretically consistent insights but may not fully represent real incinerator behaviour, including transient combustion, flame instability, emissions, slagging, or ash melting phenomena.

### ANN and contour plots

**Fig. 2:**
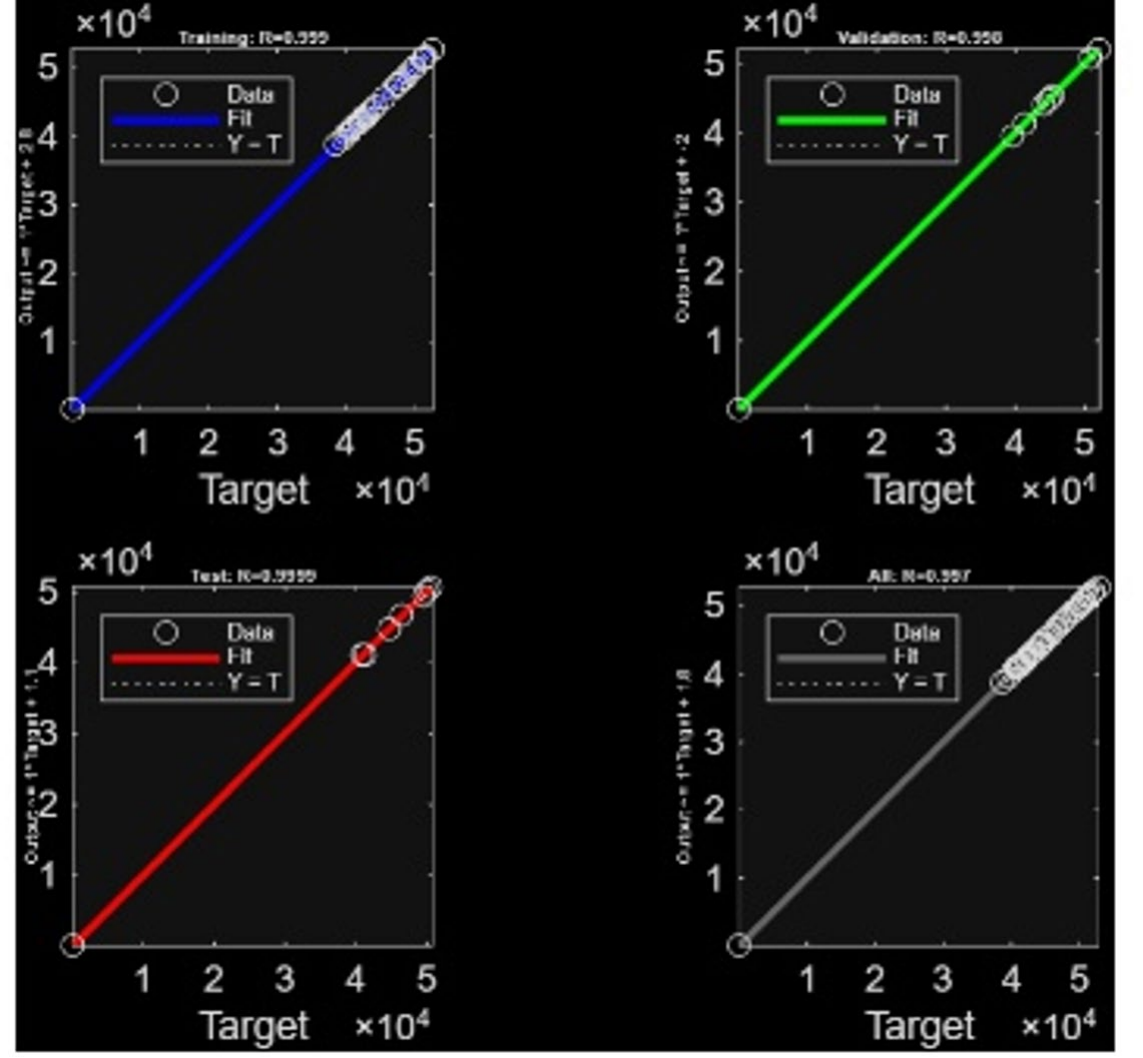
Regression plots indicating the good fit of ANN to training, testing, and validation.

The artificial neural network (ANN) model, developed with a single hidden layer of ten neurons and trained using the Levenberg-Marquardt (L-M) algorithm, was able to accurately predict both auxiliary energy requirement and efficiency of the BMW incineration process. A single 70-15-15 data split was adopted in this work, and model robustness was evaluated by comparing performance metrics across the training, validation, and testing phases, respectively. Formal k-fold cross-validation or external dataset testing was not performed due to the limited size of the experimental dataset. The tansig transfer function enabled the capture of nonlinear input-output dependencies, providing a robust mapping between the six process variables (cellulose, plastics-PE, PVC, tissue, moisture, and ash) and the two responses. The average runtime for the ANN model generation was about 36.2 s. The ANN demonstrated exceptional predictive performance, with a coefficient of determination (R^2^) of 0.999 consistently achieved across training, testing, and validation phases. This indicates near-perfect agreement between the predicted and experimental values, confirming the suitability of the model as a surrogate framework for further optimization. A mean squared error of 2e-3 ensured that the ANN model was a good fit to the data. Figure 2 indicates the goodness of fit of the training, testing, and validation dataset.

**Fig. 3:**
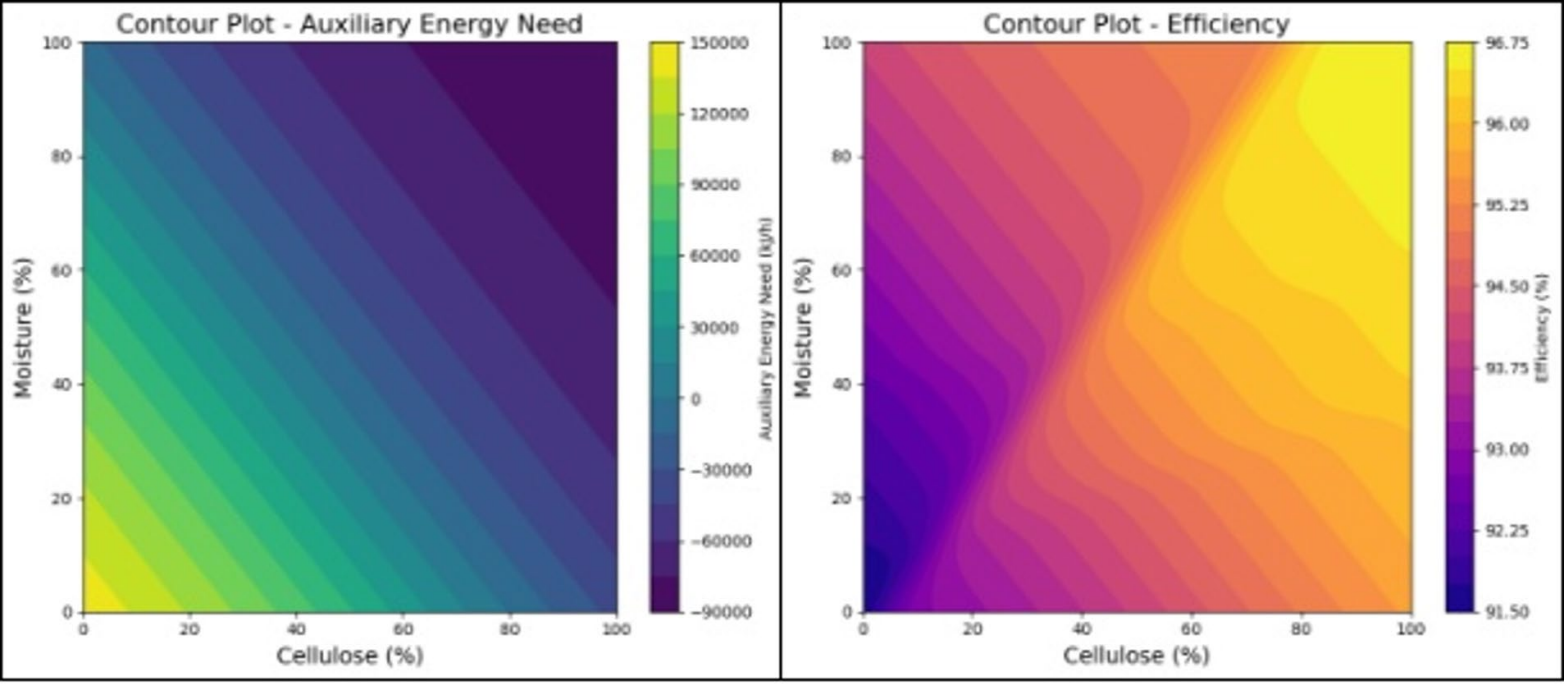
Contour plots of (**a**) Auxiliary Energy Required (kJ/h) and (**b**) Efficiency (%) of the machine derived from the ANN model.

To visualize the complex interactions among dominant process factors, 2D contour plots were constructed using ANN-predicted outputs. The contour plots generated using the ANN predictions illustrate the variation of Auxiliary Energy Requirement (Fig. 3 (a)) and Efficiency (Fig. 3 (b))with respect to cellulose and moisture content, while holding other components constant. The Auxiliary Energy plot shows a decreasing trend as both cellulose and moisture levels increase. This indicates that the ANN identified a correlation where higher cellulose and moisture reduce the predicted auxiliary energy demand. However, at the higher ends of these variables, the model extrapolates into unrealistic zones, producing values that are not physically meaningful. The Efficiency plot shows a ridge-like region of maximum efficiency (96.5–96.7%) when cellulose and moisture are both around 40%. This apparent extremum emerges from the ANN capturing interaction effects between cellulose and moisture during training. From a purely mathematical standpoint, the network identifies this region as a local optimum for maximizing efficiency.

### MOPSO

**Fig. 4:**
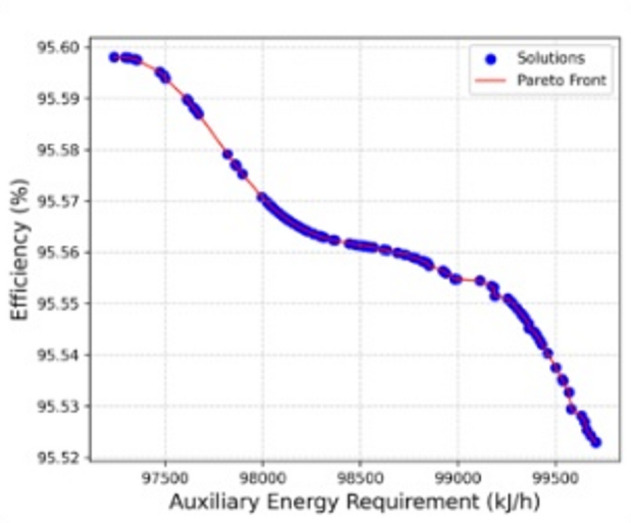
Pareto front of auxiliary energy requirement against efficiency obtained from the MOPSO results.

The application of the MOPSO algorithm to the six input variables (cellulose, plastics PE, PVC, tissue, moisture, and ash) produced a well-defined Pareto set (Fig. 4) that describes the competing objectives of minimizing auxiliary energy requirement and maximizing efficiency. The optimization algorithm, executed in MATLAB 2024b, obtained a converged set of solutions in 64.7 s. The obtained solutions exhibit a narrow but distinct band of trade-offs, where the auxiliary energy demand ranged between approximately 99.7 MJ/h at the higher end and 97.2 MJ/h at the lower end, while the corresponding efficiency values varied from about 95.52% to 95.60% and reached as high as 95.60–95.60% in select cases. Although the Pareto front appears visually compact due to the narrow numerical range of the two objectives, it in fact represents multiple feasible non-dominated solutions obtained from the MOPSO algorithm. The apparent collapse arises from the high efficiency of all optimal points, where the differences between solutions are within 0.1% in efficiency and less than 3 MJ/h in auxiliary energy. This narrow front is thus a reflection of the strong coupling between the objectives rather than a lack of diversity.

A notable outcome from the optimization is that marginal reductions in auxiliary energy input are consistently accompanied by incremental gains in efficiency. For instance, when the auxiliary energy decreased from 99.7 MJ/h to about 98 MJ/h, the efficiency improved from 95.52% to approximately 95.57–95.58%. Further decreases in energy requirement down to the vicinity of 97.2 MJ/h were associated with efficiencies approaching 95.59–95.60%. This confirms the robustness of the optimization framework in balancing the energy-efficiency objectives, and highlights the capability of the ANN-MOPSO hybrid approach in identifying non-intuitive operating regimes.

The distribution of solutions suggests that moisture and ash fractions exert a decisive influence on both objectives. The optimized results indicate that modest variations in ash (8.0 to 9.2%) and moisture (8.0 to 9.2%) shift the system closer to the Pareto frontier, with lower auxiliary fuel demand and higher efficiency. On the other hand, the values of cellulose, plastics, and tissue remained largely fixed in the optimal set (10%, 2%, and 20%, respectively), implying that the model converged toward these as stable optimum conditions rather than trade-off variables. The narrow adjustment range of PVC (2.0 to 2.3%) further emphasizes its relatively minor role compared with the dominant contribution of moisture-ash interactions. To provide practical interpretability, a reference operating point was extracted from the Pareto optimal solution set using the minimum distance to ideal solution criterion^40^. The selected operating condition corresponds to cellulose = 10%, plastics (PE) = 2%, PVC = 2.1%, tissue = 20%, moisture = 8.6%, and ash = 7.3%, which yielded an auxiliary energy requirement of 97.3 MJ/h and an efficiency of 95.59%. This point represents an optimal trade-off between both objectives and may serve as a practical operational guideline for real-world incineration scenarios where both energy efficiency and fossil fuel use are critical constraints.

From a process standpoint, this behavior is consistent with combustion dynamics in BMW incineration. Elevated moisture content typically requires greater auxiliary fuel input to drive off water, thereby lowering thermal efficiency. However, within the optimized ranges, a balanced level of moisture together with moderate ash content appears to stabilize combustion, leading to reduced fuel demand and increased efficiency. The MOPSO framework was therefore instrumental in revealing this subtle but important interplay, which would be difficult to identify using classical parametric studies.

While the ANN contour suggests a maximum efficiency at high cellulose-moisture (40–40%), the Multi-Objective Particle Swarm Optimization (MOPSO) results consistently locate the optimum in the low-cellulose (10%) and moderate-moisture (20%) region, with realistic efficiencies around 95.5–95.6%. The ANN indeed identified a ridge of maximum efficiency at high cellulose-moisture combinations, which represents a mathematical optimum within the trained model space. However, this region extends beyond the physically valid experimental domain, as also noted in the text. The ANN did not perform extrapolation during training or prediction; all inputs were confined to the experimental domain. However, when generating contour plots, the network surface was visualized over a continuous range, which may appear to extend into less-sampled regions (e.g., high cellulose-moisture combinations). These are not extrapolated predictions but graphical interpolations of the trained model surface. Unlike ANN, the MOPSO operates within feasible design and process constraints, penalizing unrealistic compositions. This ensures that optima are identified only in physically valid regions. Hence, the MOPSO algorithm explicitly enforces feasibility constraints and therefore converged toward low-cellulose and moderate-moisture compositions that minimize auxiliary energy under realistic operating conditions. The apparent divergence between ANN and MOPSO thus reflects the transition from data-driven extrapolation to physically constrained optimization rather than a modeling inconsistency. It should be noted that the ANN model was trained and validated exclusively on data generated from material and energy balance simulations. No external experimental or field-scale validation was performed in this study. Therefore, while the model demonstrates high internal predictive accuracy, its generalizability to real-world incineration systems remains to be experimentally verified.

Overall, the ANN-MOPSO hybrid strategy successfully delineated the Pareto-optimal front for the incineration process. The results confirm that a reduction of auxiliary fuel requirement is achievable without compromising efficiency, provided that the waste composition is maintained within the narrow optimal band identified by the algorithm. These findings reinforce the potential of swarm-based multi-objective optimization, when coupled with neural network surrogates, to provide actionable insights into the sustainable management of BMW incineration systems.

### Limitations of the study

This study is based on a theoretical framework and simulated data derived from heat and material balance calculations rather than experimental measurements. While this approach allows systematic control of process parameters, it may not fully capture the variability of real biomedical waste composition or combustion dynamics. The compositional variables form a constrained mixture (summing to 100%), which introduces multicollinearity among predictors; consequently, the ANOVA results should be interpreted as indicative trends rather than fully independent effect estimates. The ANN model, though exhibiting high accuracy (R^2^ > 0.99), is susceptible to overfitting when trained on limited datasets. To minimize this risk, the dataset was divided into training, validation, and testing subsets in a 70:15:15 ratio, ensuring independent performance evaluation. The findings are based solely on simulated data derived from theoretical heat and material balance models. No external experimental validation or cross-dataset testing was conducted. As such, the current MOPSO results should be considered theoretically indicative rather than empirically confirmed. Future work will incorporate laboratory-scale incineration trials and field validation to assess real-world applicability. The energy balance formulation assumes steady-state conditions, complete combustion, and a fixed radiative loss fraction (5%), which may differ under practical operating conditions. This study is purely simulation-based, relying on heat and material balance calculations without experimental data or physical validation. While the approach enables conceptual feasibility assessment, trend identification, and optimization, it does not account for combustion instability, pollutant formation, operational constraints, or equipment-level behaviour. Therefore, the results should be considered theoretically indicative rather than empirically confirmed. These limitations are acknowledged as part of the study’s theoretical scope, and future work will include experimental validation under variable feed and operational conditions to assess model robustness and general applicability.

## Conclusions

The integrated experimental-computational investigation confirmed that waste composition decisively affects the energy performance of biomedical waste incineration. The cellulose fraction reduced auxiliary LPG demand from 33.8 to 27.4 kg/h, while higher tissue and moisture contents increased fuel consumption and lowered thermal efficiency from 95.6% to 94.8%. ANOVA verified cellulose and moisture as the dominant parameters, with plastics, PVC, and ash showing minor influence. The developed ANN model reproduced all experimental results with high accuracy (R^2^ > 0.99), and its coupling with MOPSO identified an optimal combination of 10% cellulose, 20% moisture, and 8–9% ash, achieving an auxiliary energy demand of 97.2 MJ/h at 95.6% efficiency.

Unlike previous studies that applied independent empirical or optimization models, this work uniquely integrates experimental thermodynamic analysis with an ANN-MOPSO hybrid, demonstrating its capability to capture nonlinear composition-performance relationships within a small dataset. The framework offers a transferable approach for minimizing fossil-fuel use in BMW incineration, with future extensions possible toward emission quantification and life cycle assessment for broader sustainability evaluation.

## Data Availability

The data may be made available from the corresponding author upon reasonable request, as it is a part of an ongoing project.
